# Supplementary material for: Examining representation of women in leadership of professional medical associations in India
Source: PLOS Glob Public Health. 2024 Aug 12;4(8):e0003587. doi: 10.1371/journal.pgph.0003587 (PMC11318910; doi:10.1371/journal.pgph.0003587)
Supplement: S1 Text — (DOCX) [file pgph.0003587.s002.docx]

# **Supporting Information**

**Reflexivity Statement**

The author team consists of five Indian origin researchers- four women and one man. They have diverse disciplinary backgrounds, research experiences, and are at different stages of their career. The first author is an early-career researcher with academic background in public health and research interests around gender equity, health systems, and injury prevention. The second author is a mid-career researcher with an academic background in public health, with a particular focus on health policy and systems research. She holds experience of extensive research on the role of PMAs in shaping health policies in India. The third author is a mid-career public health professional and a medical doctor by training, with research and professional interests in healthcare quality and patient safety. The fourth author is a social scientist with extensive experience in gender mainstreaming theory and practice and a focus on gender equality and social inclusion research in health. The last author is a mid-career researcher and a medical doctor trained in India with research interest in health policy and systems, especially health workforce governance, and role of actor power in health policy making.

Four of the authors are not members of any Professional Medical Association in India. One author is a life member of the Indian Association of Preventive and Social Medicine (IAPSM) and Indian Public Health Association (IPHA) with voting rights to elect leadership committees of these associations. Two authors are members of the Women in Global Health, India chapter. None of the authors are a member of the Indian Medical Association (IMA). One author was engaged with PMAs in the past for professional work, but she is not currently and has not been part of any PMA.
