# Supplementary material for: Examining representation of women in leadership of professional medical associations in India
Source: PLOS Glob Public Health. 2024 Aug 12;4(8):e0003587. doi: 10.1371/journal.pgph.0003587 (PMC11318910; doi:10.1371/journal.pgph.0003587)
Supplement: Attachment — Submitted filename: Response to reviewers.docx [file pgph.0003587.s003.docx]

08 January 2024

To,

The Editor-in-Chief,

PLOS Global Public Health

**Subject:** Rebuttal letter in support of manuscript ID PGPH-D-23-02642

Dear Prof Madhukar Pai,

We are pleased to submit our revised manuscript titled ‘**Examining representation of women in leadership of professional medical associations in India**’ [Manuscript PGPH-D-23-02642] for potential publication in PLOS Global Public Health, following the editor's feedback on the original submission [PGPH-D-23-01195]. Our paper addresses an area where little to no research has been conducted in India and similar contexts – the role of women in leadership position in representative medical organizations. This is an issue that has received some attention in high-income settings, but very little (to none) in Global South contexts, including India. This topic connects strongly with efforts to “*increase diversity and inclusion in public health*” (one of PLOS GPH’s goals), with recent attention to the lack of gender diversity in global health organizations, editorial boards of academic journals, and health worker associations globally, including several important pieces in this journal.

The editor has noted that we do not meet the criteria due to “the lack of a strong argument, insufficient methodological details, and a weak conclusion that does not seem well-aligned with the study's findings”. As per our understanding of the criteria, we appear to have met each of the seven criteria for publication in the journal. In terms of the criterion that we presume is being raised by the editor (‘Experiments, statistics, and other analyses are performed to a high technical standard and are described in sufficient detail’), we have followed a similar approach to highly cited papers, such as Waseem et al (2020) and Bhaumik & Jagnoor (2019). In addition, we have tried to address the editor's concerns, and have focussed on strengthening our argument and enhancing the alignment of our conclusion with the study's findings.

While we appreciate Reviewer 1's constructive feedback, we contest Reviewer 2's viewpoint that research on gender representation lacks value due to its global recognition as a problem. For example, the Reviewer states that *“…This is a known fact. I think this is not new as the same applies to the rest of the positions of national significance in most countries, India inclusive. This applies to several parliaments, ministerial appointments and the like around the world and definitely in India, then what would be expected to turn out differently with respect to the MPAs?*” We strongly push back on this reasoning as grounds for rejecting the paper and firmly believe our study contributes a nuanced perspective relevant to the specific context of medical associations in India.

We kindly request a reconsideration of the decision, advocating for a re-review, and remain committed to working collaboratively to address any remaining concerns for a successful publication.

Thank you for your time and consideration of this manuscript and we look forward to hearing from you.

Yours sincerely,

Pratishtha Singh

[Pratishthasingh97@gmail.com](mailto:Pratishthasingh97@gmail.com)

(On behalf of Veena Sriram, Sonali Vaid, Sharmishtha Nanda, Vikash R. Keshri)
