# Supplementary material for: Examining representation of women in leadership of professional medical associations in India
Source: PLOS Glob Public Health. 2024 Aug 12;4(8):e0003587. doi: 10.1371/journal.pgph.0003587 (PMC11318910; doi:10.1371/journal.pgph.0003587)
Supplement: Attachment — Submitted filename: Response to Reviewers.docx [file pgph.0003587.s004.docx]

Dear Dr Hammad,

We thank you and the Reviewers for your time and review of this article and are grateful for the recognition of the importance of this work. Request you to kindly refer to the below table for our response to all comments by Reviewers.

| **Reviewer 1** | **Authors’ response** |
| --- | --- |
| This is an important contribution to human resources in health. It would be useful if the authors indicate the proportion of women in the major specialisations. This would help to support their argument that women are underrepresented in certain specialisations and overrepresented in others. How does this get reflected in their presence in professional organisations? | We thank Reviewer 1 for their time and comments. To address their point, we have included the following text in the Introduction section:  *‘Further, while limited data is available regarding the proportion of women in medical specializations, available information suggests low levels of representation [29, 30].’* |
| **Reviewer 2** | **Authors’ response** |
| Strengths  1. strong arguments  2. well written | We thank Reviewer 2 for their positive feedback. |
| weakness  1. long introduction which can be shorten | The introduction has now been shortened to make it crisp. Some parts have been reframed and the following statements have been removed-  ‘*Recognizing the deep and persistent inequities that currently exist in health outcomes and access to health services, the inclusion of women and gender-diverse people in policy processes and decision-making is therefore fundamental to ensuring progress*.’  ‘*Globally, international, regional, and national PMAs play a critical role in regulating medical education and practice, setting standards, advancing various dimensions of practice and ethics, influencing health policy prioritization, and engaging in policy development and implementation.*’  ‘*PMAs have on occasion advocated and fought for the interest of the public and patients [22, 23], and at other times have focused on safeguarding their own rights and safety [24, 25].’*  ‘*Understanding these factors are crucial to identifying and addressing a range of systemic challenges, including those faced by women physicians who may experience discrimination within the Indian health sector, and might also contribute to a broader understanding of the reasons behind the lack of a well-integrated gender focus in health policies and programs in the Indian health sector.*  *The currently available literature on gender representation in PMAs are limited and are largely from high-income countries (HICs).*’ |
| 2, lots of repetitive arguments and statements | Thank you for this comment. As noted above, we have sought to reduce repetitions throughout the manuscript. |
| 3. will recommend a graph indicating the changes over the decades | We agree that adding such a graph would be valuable. However, this is outside the scope of the current manuscript. Our initial data extraction did not include longitudinal collection of data across decades, and thus implementing this suggestion would again require additional data extraction and analysis that would be outside the scope of our current analysis. |
| 4. Discussion should include cultural and social roles of women what could be contributing to a leaky pipeline | The first paragraph of the Discussion section has been amended to include the following-  ‘*This underrepresentation is linked to prevalent gender stereotypes and discrimination, unconscious favouring of men in decision-making and leadership roles, and a lack of networking opportunities. Further, social and cultural expectations related to motherhood and caregiving such as the pressure to prioritize family responsibilities over career advancement, limited support for work-life balance, and societal norms that undervalue women's professional contributions, significantly contribute to the barriers women face in advancing their careers.’* |
| 5. any comparison with any other Asean country data, allied health organizations including nursing and radiographers? | We weren’t able to find any information for gender breakdown of leadership in PMAs of ASEAN countries. We have tried to include all data that is relevant to the health sector in India. |
| **Reviewer 3** | **Authors’ response** |
| A very well written manuscript presenting important data with an excellent discussion in context.  Limitations are discussed however suggest to expand discussion of the sampling method of the societies reviewed - how did the authors ensure that the societies in the sample are representative and how confident are they that the results can be generalized to all local/national societies | We thank Reviewer 3 for their suggestion on generalizability of the sampling method. We have added the following text in the Limitations section-  ‘*In addition, while we included PMAs from all broad specializations, we only selected certain super-specialty associations based on their engagement in policy and practice. Therefore, though our findings can be generalized at the local and national level to the broad specialty categories, but not to super-specialties.’* |
| **Reviewer 4** | **Authors’ response** |
| I applaud the authors on this insightful manuscript with addresses gender disparities in leadership from a LMIC perspective.  Suggest the authors to elaborate on the 32 sub chapters of the Indian Medical Association to provide more context to readers who are familiar with the organizational structures of Indian PMAs. | We thank Reviewer 4 for their feedback and comments.  In the Methods section, the paragraph around the IMA has been edited to say-  *‘The IMA is the largest PMA in India, with a membership of around 3,50,000 modern medicine doctors. To allow for decentralized decision-making, it has sub-chapters in all 28 states and four Union Territories of India. These institutions play a critical role in shaping medical practices and dictating healthcare policies and priorities within their respective regions. In addition to the above, we also analysed the leadership of the sub-chapters of IMA by extracting data on the gender of the presidents and secretaries*.’ |
| May the authors justify in the methodology why an assumption was made regarding gender for this specific study. ( When gender is not identified, assumption was male by default. | Though our Limitations section mentions that this assumption was made only for approximately 3% of entire sample, we have amended text in the Methods section to say the following-  *‘For the small number of people who had no internet presence, or their gender could still not be determined, the person was assumed to be a man, based on the rationale that women typically have gender signifiers in front of their names and that the field of medicine is overwhelmingly male.’* |
| Line 169- Is this meant to be 350 000 or 3 500 000 | As per the [website of the Indian Medical Association](https://www.ima-india.org/ima/free-way-page.php?pid=2), they have a membership of 3.5 lakhs doctors, i.e. 350,000. |
| Line 179- There is reference to lifespan of associations - did some dissolve? If not, suggestion is to rephrase to make it clearer. | The sentence has been reframed to say-  *‘Out of 46 PMAs, 24 have been in existence since are more than 50 years old, while the average age and the average lifespan of the included PMAs is 49 years.’* |
| The authors provided results of current and past leadership which is a very important aspect of the study. Considering the oldest association is 95years, is it possible to review the trend for each decade regarding the number of women in leadership. It will be enlightening to note if overall there is an improvement or rather stagnation to guide interventions that assist in promoting women participation in leadership roles overall. | As we have mentioned in response to Reviewer 2, we agree that adding such a graph would be valuable, however, this is outside the scope of the current manuscript. Our initial data extraction did not include longitudinal collection of data across decades, and thus implementing this suggestion would again require additional data extraction and analysis that would be outside the scope of our current analysis. |
| Do we also have the number of women who are members in some of the associations referenced specifically from India. They could use data for IMA only for example, or obstetric and gynecology association to obtain the proportion of women who are members.  Overall, an important and crucial topic that overall can improve health outcomes in this population . | Though we agree that this data would be valuable to add, breakdown of demographic indicators, such as gender are not publicly available for IMA or FOGSI. |

We hope we were able to address all Reviewer concerns. In addition to adding this text, we’ve made minor alterations to some of the existing content of the article to flow better and make it more compelling. Lastly, we have also resubmitted the two figure files in accordance with the PACE guidelines.

Once again, we thank you for your time and feedback. We request your kind consideration for publication of this manuscript.

Yours sincerely,

Pratishtha Singh

[Pratishthasingh97@gmail.com](mailto:Pratishthasingh97@gmail.com)

(On behalf of Veena Sriram, Sonali Vaid, Sharmishtha Nanda, Vikash R. Keshri)
